# [Retracted] Kevetrin induces apoptosis in *TP53* wild-type and mutant acute myeloid leukemia cells

**DOI:** 10.3892/or.2026.9154

**Published:** 2026-06-22

**Authors:** Roberta Napolitano, Serena De Matteis, Silvia Carloni, Samantha Bruno, Giulia Abbati, Laura Capelli, Martina Ghetti, Maria Teresa Bochicchio, Chiara Liverani, Laura Mercatali, Daniele Calistri, Antonio Cuneo, Krishna Menon, Gerardo Musuraca, Giovanni Martinelli, Giorgia Simonetti

Oncol Rep 44: 1561–1573, 2020; DOI: 10.3892/or.2020.7730

Subsequently to the publication of the above article, a concerned reader drew to the Editor's attention that, regarding the control β-actin blots shown in Fig. 5A for the MOLM-13 cell line, the blots featured for the first and the second lanes in the gel slice were strikingly similar, suggesting that this figure may have potentially undergone inappropriate editing.

After having evaluated these data independently in the Editorial Office, the concerns of the reader were shown to have been well-founded. Therefore, in view of the uncertainties regarding the assembly of the western blot data in this figure, the Editor of *Oncology Reports* has decided that this paper should be retracted from the Journal on account of a lack of confidence in the presented data. The authors were asked for an explanation to account for these concerns, but the Editorial Office did not receive a satisfactory reply. The Editor apologizes to the readership for any inconvenience caused.

